# Evaluation of the Nutritional, Rheological, Functional, and Sensory Properties of Cookies Enriched with Taro (*Colocasia esculenta*) Flour as a Partial Substitute for Wheat Flour

**DOI:** 10.3390/foods14203526

**Published:** 2025-10-16

**Authors:** Sylvestre Dossa, Christine Neagu, Dacian Lalescu, Monica Negrea, Daniela Stoin, Călin Jianu, Adina Berbecea, Liliana Cseh, Adrian Rivis, Mariana Suba, Ersilia Alexa

**Affiliations:** 1 Faculty of Food Engineering, University of Life Sciences, “King Mihai I” from Timisoara, Aradului Street No. 119, 300645 Timisoara, Romania; dossasylvestre@usvt.ro (S.D.); christine.neagu@usvt.ro (C.N.); monicanegrea@usvt.ro (M.N.); danielastoin@usvt.ro (D.S.); calinjianu@usvt.ro (C.J.); adrianrivis@usvt.ro (A.R.); ersiliaalexa@usvt.ro (E.A.); 2 “Food Science” Research Center, University of Life Sciences “King Mihai I” from Timisoara, Aradului Street No. 119, 300645 Timisoara, Romania; 3 Faculty of Agriculture, University of Life Sciences “King Mihai I” from Timisoara, Aradului Street No. 119, 300645 Timisoara, Romania; adina_berbecea@usvt.ro; 4 Romanian Academy, “Coriolan Dragulescu” Institute of Chemistry, Mihai Viteazu No. 24, 300223 Timisoara, Romania; lcseh@acad-icht.tm.edu.ro (L.C.); marianasuba@gmail.com (M.S.)

**Keywords:** *Colocasia esculenta*, nutritional, phytochemical, rheological, functional biscuit

## Abstract

This study evaluated the impact of incorporating taro flour (*Colocasia esculenta*) into wheat-based biscuit formulations, focusing on nutritional, phytochemical, rheological, technological, and organoleptic characteristics. Four distinct types of biscuits were formulated with 0% (CC), 10% (TWC1), 20% (TWC2), and 30% (TWC3) taro. The results indicate that taro flour (TF) has a higher moisture, ash, and gallic acid content, as well as significant mineral richness, particularly in potassium, magnesium, and calcium, compared to wheat flour (WF). It has been shown that the gradual incorporation of TF (10 to 30%) into composite flours improves the bioavailability of certain micronutrients and polyphenols, while maintaining a harmonious balance with the flavonoids characteristic of wheat, such as quercetin. The evaluation of antioxidant activity indicates a higher value for TF (50.71%) compared to WF (36.53%), with a significant increase in activity observed in enriched cookies (58.92% for TWC3 vs. 31.36% for CC). In addition, the incorporation of taro flour modifies the technological properties of the cookies, resulting in a decrease in thickness and baking yield, as well as an increase in diameter and spread rate. This modification may result in a crisper texture. However, a high substitution rate (30%) resulted in a decrease in dough stability and baking yield due to a reduction in gluten and a change in dough structure. In terms of antinutritional profile, partially replacing wheat flour with taro flour significantly reduces phytic acid content, while moderately increasing oxalate content. Sensory analysis of different types of cookies indicated that moderate substitution levels (10%) tended to improve or maintain sensory quality, while higher substitution levels (20% and 30%) tended to reduce overall acceptability. Linear correlations showed a positive relationship between antioxidant activity and polyphenols and minerals, but a negative relationship with proteins and specific technological properties. In summary, the incorporation of taro flour into cookie formulations represents a promising strategy for improving the nutritional value and functional potential of baked goods without compromising their technological quality. These results confirm the value of taro as a functional ingredient that can contribute to the development of healthy foods.

## 1. Introduction

In the contemporary context, the baking industry is actively exploring alternatives to wheat flour to produce foods with high nutritional value that are gluten-free for individuals with gluten allergies. This approach presents several promising prospects for the future of the agri-food industry, including the partial or total substitution of wheat flour by other flours that are more nutritious [1].

Taro (*Colocasia esculenta*) is a traditional crop with significant nutritional and pharmaceutical potential when compared to other tuber crops. It is a root crop that is extensively cultivated in tropical regions, including Southeast Asia, the Pacific Islands, Africa, the United States, and the Mediterranean. It has been identified as a promising candidate for the future of innovative food production, particularly within the context of underutilized roots and tubers. This initiative aims to address the pressing issues of chronic malnutrition and hidden hunger that are prevalent in Asia [2,3]. Taro is utilized as a subsistence crop in particular developing countries, thereby underscoring its pivotal function in ensuring food security [4]. It offers significant opportunities for dietary diversity and sustainability of food production systems in developing and low-income countries, due to its nutritional density and ability to adapt to low-input agricultural systems with greater adaptability. Taro is also a crop of potential interest to the agri-food industry. The material’s versatility in processing allows for its transformation into a diverse range of products. A thorough market analysis reveals the presence of various food products derived from taro. The aforementioned products include chips, starch, taro flour, taro paste, taro cookies, and noodles. Moreover, it is a pivotal component in a diverse array of infant food products, attributable to its elevated nutritional value. In this context, ref. [5] reported that the use of taro powder as an ingredient in infant foods has recently attracted interest. Researchers who have utilized taro as a food ingredient in canned products and extruded paste products have reported that taro is also processed into products such as packaged poi, jarred poi (baby food), dehydrated poi, and fried taro chips [6].

Research has demonstrated the efficacy of incorporating taro flour as a partial substitute for wheat flour in bread-making, with notable benefits including the reduction in developing countries’ reliance on wheat imports and, more crucially, the reduction of production costs. This substitution has been shown to enhance the nutritional value of baked goods [7,8,9,10,11]. Despite taro’s nutritional richness, potential, status as one of the oldest domesticated plants, and the diversity of its derivatives, it contains antinutritional factors. These include phytates, cyanogenic glycosides, tannins, amylase inhibitors, and oxalates. These elements can pose health risks and affect the bioavailability of the nutrients that taro is rich in [12]. In addition, research on the use of taro flour as a substitute for wheat flour in baked goods such as bread and cookies remains limited, as it neglects the antinutritional components of taro [7,12,13,14].

Cookies are one of the most widely consumed baked goods worldwide due to their palatability, convenience, and long shelf life. Therefore, they would be an ideal medium for nutritional supplementation with protein, fiber, and other functional ingredients [15,16]. In this context, this study aims to evaluate the nutritional, rheological, functional, and sensory properties of cookies enriched with taro flour (*Colocasia esculenta*) as a partial substitute for wheat flour. Also, the antinutritional compounds such as phytic acid and oxalates were determined in order to assess the potential negative impact of consuming flour-based products made from taro on health.

## 2. Materials and Methods

### 2.1. Preparation of Taro Flour

**Figure 1:**
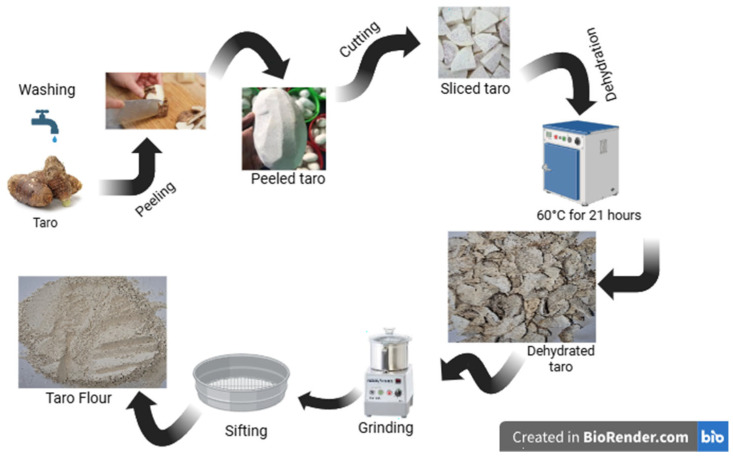
Technological flow for obtaining taro flour. Figure created with BioRender.com, accessed 2 July 2025.

The processing of taro tubers (Figure 1) was carried out by the methods described by [7,9], with some adjustments. Following the harvesting of the taro tubers, they were meticulously washed with purified tap water. The taro tubers were meticulously peeled by hand and sliced into pieces approximately 0.5 cm thick. The slices were then dehydrated in an oven at 60 °C for 21 h. The dried slices were subsequently ground using a knife mill, which grinds materials into a flour with a particle size of less than 30 mesh. The objective of this operation was to obtain a flour of uniform size. The taro flour (TF) thus obtained was stored in polypropylene bags and subsequently placed in an airtight container. The sample was stored in a dry environment until its subsequent use.

### 2.2. Preparation of Composite Flours

Taro tubers were purchased from Beninese producers and processed into flour, while type 000 wheat flour was purchased from the Profi supermarket in Romania. Three types of taro/wheat flour blends were formulated according to [7]. The formulated flour blends were TWF1 (10% taro flour (TF) and 90% wheat flour (WF)), TWF2 (20% TF and 80% WF), and TWF3 (30% TF and 70% WF).

### 2.3. Analysis of the Rheological Parameters of Different Flours

**Figure 2:**
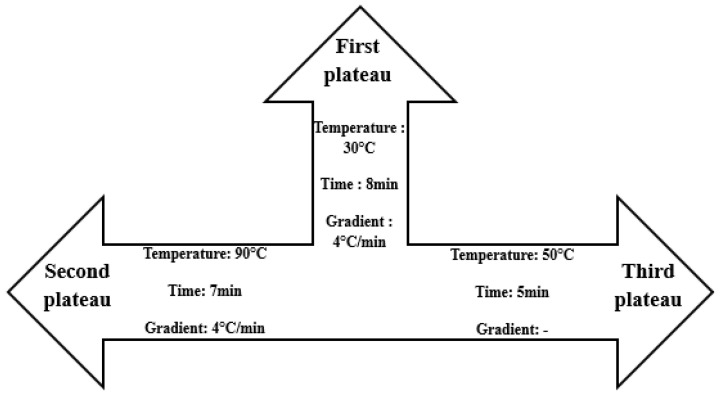
Different characteristics of the different Mixolab plateaus.

To determine the effect of partially replacing wheat flour with taro flour on the rheological properties of the different flour samples, a rheological analysis was carried out according to the “Chopin+” protocol of the Chopin Mixolab [17]. To do this, it was first necessary to determine the moisture content of the sample using a thermobalance (Kern & Sohn GmbH, D-72336 Balingen, Germany). Respectively, 46.29, 46.07, 44.74, and 44.84 g of samples WF, TWF1, TWF2, and TWF3 were weighed and placed in the Mixolab bowl. There were three bowls, the characteristics of which are shown in the Figure 2. The mixing speed was 80 rpm.

### 2.4. Cookie Preparation

**Figure 3:**
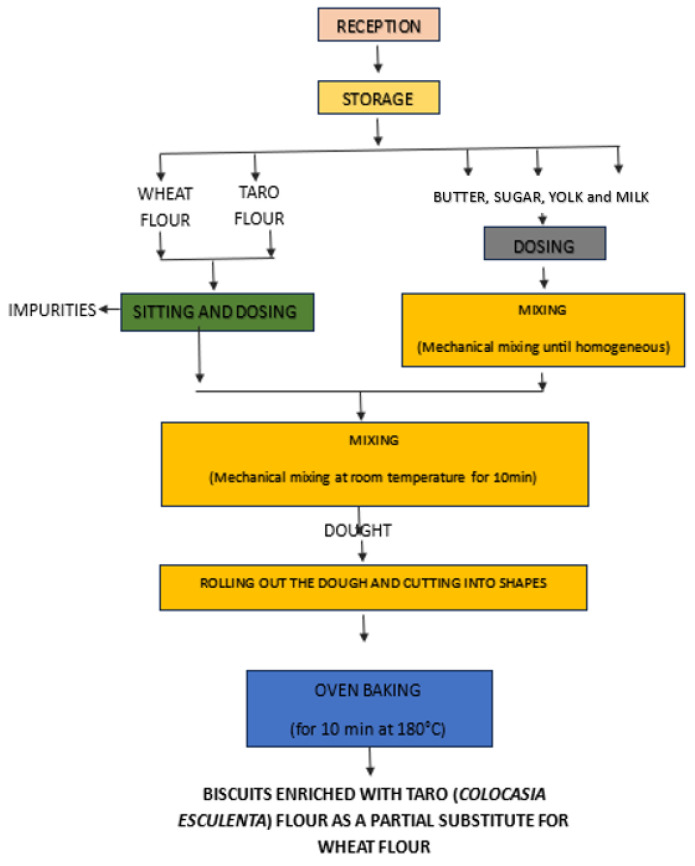
Technological process for obtaining wheat biscuits enriched with taro.

The cookies were prepared according to [18,19] with a few modifications. The sugar, salt, butter, and egg used in the formulation of the different cookies were purchased at the Auchan supermarket in Timisoara, Romania. Four (4) types of cookies (CC, TWC1, TWC2, and TWC3) were formulated with different levels of WF substitution by TF (CC-control cookie with 100% WF; TWC1-cookie with 10% TF and 90% WF; TWC2-cookie with 20% TF and 80% WF; TWC3-cookie with 30% TF and 70% WF. The production protocol is shown in the Figure 3.

The various biscuits obtained and the composition of the ingredients used in their formulation are presented in the Table 1.

### 2.5. Determination of Proximate Composition

As part of this study, the immediate composition of the various flours (taro flour, wheat flour, and various blended flours) was determined. This methodology was applied systematically to all of the cookie samples obtained. The methodologies used to achieve these objectives are set out in the Table 2.

### 2.6. Determination of Macro- and Microelements in Different Samples of Flour and Cookies

The determination of the macro- and microelement content of the different samples analyzed in this study was carried out according to the methodology established by Plustea et al. (2022) [23]. The results are expressed in milligrams per kilogram (mg/kg).

### 2.7. Determination of Phytochemical Profile

#### 2.7.1. Preparation of Alcoholic Extracts

The extraction of phytochemical compounds was carried out in accordance with the procedure described by Dossa et al. (2023) [24]. For this purpose, 1 g of each sample was dissolved in 10 mL of 70% ethanol in a hermetically sealed container. The resulting solution was then shaken for 30 min and filtered.

#### 2.7.2. Evaluation of Total Phenolic Content (TPC), Antioxidant Activity (AA), and Individual Polyphenols by HPLC

The analytical methods used for the determination of total phenolic content (TPC), antioxidant activity (AA), and individual polyphenols by HPLC is presented in Table 3.

### 2.8. Physical Analyses Performed on the Cookies

The physical analyses performed on the different cookies obtained were based on the methods described by [7] with some modifications. The parameters determined were baking yield, thickness increase, diameter increase, and spread ratio (D/T).

#### 2.8.1. Determination of Baking Yield

The baking yield was determined by dividing the weight of the baked cookies by the weight of the unbaked cookies [7].

#### 2.8.2. Diameter Increase

The diameter increase, expressed as a percentage, is calculated using the following formula: (final D − initial D)/(initial D) × 100, where final D and initial D represent the final and initial diameters, respectively, and the result is rounded to the nearest hundredth [7].

#### 2.8.3. Thickness Increase

The thickness increase was evaluated by calculating the difference between the thickness measurements before and after baking [7].

#### 2.8.4. Spread Ratio (D/T)

The spread ratio (D/T) was determined using the ratio between the elongation (diameter) and the thickness of the baked cookies [28]. As part of the experiment, ten cookies from each formula, in triplicate, were randomly selected to evaluate the physical properties, representing a total of 30 cookies.

### 2.9. Fourier Transform Infrared Spectroscopy (FTIR) of Composite Flours

For Fourier transform infrared (FTIR) analysis, we used a Nicolet Is50 FT-IR instrument (Thermo Fisher Scientific, Waltham, MA, USA) equipped with an ATR crystal. Infrared (IR) spectra were obtained in the spectral range from 4000 to 400 cm^−1^, with 32 scans at a resolution of 4 cm^−1^. The procedure began with a preliminary step of heating the sample, commonly referred to as pre-drying, which consisted of exposure to a temperature of 40 °C for an entire night. The purpose of this step was to eliminate moisture that could potentially interfere with the rest of the analysis, specifically the spectroscopic analysis. Once the drying process was complete, the sample was placed directly on the ATR crystal of the FTIR spectrometer.

### 2.10. Determination of Antinutritional Factors

#### 2.10.1. Determination of Phytic Acid Content

The phytic acid concentration was determined following the manufacturer’s protocol, namely the K-PHYT Phytic Acid/Total Phosphorus Assay Kit (Megazyme, Bray, Ireland; Neogen Corporation, Lansing, MI, USA), as detailed in the most recent 2025 version of the protocol. This methodological approach, based on enzymatic and colorimetric principles, allows for the accurate quantification of total phosphorus released by phytic acid and its lower forms of inositol phosphate (IP_2_–IP5). As part of the chemical analysis, a sample of approximately 1.0 g was meticulously ground before being extracted using 20 mL of 0.66 M hydrochloric acid, with continuous stirring, for a period of 3 h at room temperature (or overnight), according to standard protocols. Following this extraction step, the mixture was centrifuged at a speed of 15,000× *g* for 10 min, allowing the removal of unwanted components and isolation of the final product. The clear supernatant was neutralized with an equal volume of 0.75 M sodium hydroxide and subjected to sequential enzymatic hydrolysis. In our experiments, the first step consisted of adding phytase (pH 5.5 buffer) to hydrolyze phytic acid into low molecular weight inositol phosphates and inorganic phosphate (Pi). Then, after a period of 10 min at a temperature of 40 °C, we introduced alkaline phosphatase (pH 10.4 buffer) to complete the dephosphorylation. The reaction was stopped with 0.3 mL of 50% (*w*/*v*) trichloroacetic acid, and the mixture was centrifuged at 13,000 rpm for 10 min. The released Pi was determined colorimetrically by forming a blue molybdenum complex through the reduction of phosphomolybdic acid in the presence of ammonium molybdate and ascorbic acid under acidic conditions. The absorbance was measured at 655 nanometers (nm) using a spectrophotometer (model Specord 205; Analytik Jena AG, Jena, Germany), and the phosphorus concentration was calculated from a calibration curve (0–7.5 micrograms [µg] of phosphorus [Pi]). The phytic acid content was then determined on the assumption that phosphorus accounts for 28.2% of the phytic acid molecule. The method is based on the colorimetric procedure originally developed by McKie and McCleary [29] and subsequently refined in the K-PHYT protocol [30].

#### 2.10.2. Determination of Oxalate Content

The oxalate content of the different flour samples (WF, TF, TWF1, TWF2, and TWF3) and biscuit samples (CC, TWC1, TWC2, and TWC3) was determined using the method described in [31], which consists of three successive steps: digestion, precipitation, and permanganate titration. As part of our experiments, 2 g of each sample was meticulously digested in 190 mL of distilled water and 10 mL of 6N HCl at a temperature of 100 °C for one hour. The volume was then precisely adjusted to 250 mL, and the sample was rigorously filtered. Next, 125 mL of the filtrate was neutralized to pH 4–4.5 using ammonia (NH_4_OH) in the presence of methyl red, heated to 90 °C, and then treated with 10 mL of 5% calcium chloride (CaCl_2_) solution to precipitate calcium oxalate. The mixture was kept at 5 °C for 12 h. The precipitate obtained was centrifuged at 2500 rpm for five minutes. It was then dissolved in 10 mL of 20% (*v*/*v*) H_2_SO_4_, and the solution thus obtained was titrated using a standardized 0.05 N KMnO_4_ solution until a pale pink color persisted for 30 s. This indicated the endpoint of the titration.

The oxalate content, expressed in mg/100 g, was determined using the following mathematical equation:

$$Oxalate\;content=\frac{V\times Vme\times Df\times 10^{5}}{Me\times Mf}$$

where V is the volume of KMnO_4_ (mL), Vme is the equivalent mass volume (0.00225 g/mL), Df is the dilution factor, Me is the molar equivalent between KMnO_4_ and oxalate, and Mf is the mass of the sample (g).

### 2.11. Evaluation of Sensory Profils

Sensory evaluation of the different cookie samples was carried out for consumer acceptance and preference by a panel consisting of 34 assessors (15 males and 19 females), non-smokers, from the ages of 19–46, without known cases of food allergies. The samples were presented in cardboard plates with three-digit characters, once at a time to each evaluator. Panelists were asked to rinse their mouths with still water between sample evaluations. The appearance, taste, flavor, texture, and general acceptability of the products were rated using a 5-point hedonic scale, where 5 represents ‘extremely like’ and 1 represents ‘extremely dislike’. All 34 panelists were trained according to ISO 6658:2017 [32].

### 2.12. Statistical Analysis

All determinations were executed in triplicate. The results are presented as the mean values ± standard deviation (SD). The differences between the mean values were analyzed using Duncan’s Multiple Range Test after ANOVA. Significant differences were considered when the *p*-values were less than 0.05. All statistical analyses were performed using R Statistical Software (v4.3.3; R Core Team 2023, Vienna, Austria).

## 3. Results

### 3.1. Determination of Immediate Composition

The Table 4 shows the results of the moisture, mineral, protein, lipid, and carbohydrate composition of the different flours and cookies.

The evaluation of the nutritional characteristics of compound flours and biscuits enriched with taro flour (TF) from the table above reveals significant variations depending on the proportions of taro flour incorporated. The moisture content of compound flours decreases as the proportion of taro increases, from 10.99% for TWF1 (flour composed of 10% taro) to 10.53% for TWF3 (flour composed of 30% taro). This trend is also found in cookies, where moisture drops from 6.63% in control cookies (CC) to 5.86% in those containing 30% taro (TWC3). This can be explained by the higher moisture content in wheat flour (11.50%) than in taro flour (10.38%). Similar results have been obtained by [1,33,34]. In fact, in their studies [1,33,34], not only was wheat flour richer in water content than taro flour, but also the increase in the proportion of taro in compound flours and finished products was also synonymous with a reduction in water content. This reduction in moisture could improve the shelf life of finished products.

The ash content, which is an indicator of mineral richness, is significantly higher in TF (2.58%) than in WF (0.28%). This difference translates into a gradual increase in ash content in composite flours as the proportion of taro increases. Indeed, the mineral content rose from 0.35% for TWF1 to 0.76% for TWF3. The same trend is observed in cookies (1.07% for CC versus 1.78% for TWC3). These results confirm that incorporating taro flour is an effective way to enrich baked goods with minerals, which corroborates previous work on the use of taro flours [1,7,33,34].

On the other hand, the incorporation of taro flour leads to a gradual decrease in protein content. Wheat flour has a protein value of 11.30%, compared to 6.76% for taro flour, resulting in a decrease of up to 9.99% in composite flours and 9.83% in biscuits enriched with 30% taro. However, this reduction remains moderate and acceptable in the context of biscuits, which are often less protein-intensive products. These results confirm those of [1,7,33,34], indicating that partial substitution of wheat flour with taro flour would reduce the protein potential of composite flours and fine products.

The lipid content varies slightly but significantly between wheat flour and taro flour (approximately 1.3%), as is the case for blended flours. However, it increases significantly in cookies (20.80–21.04% between TWC3 and CC), reflecting the addition of fat during formulation. The incorporation of taro, therefore, does not significantly affect the lipid content of either the blended flours or the final product, which is consistent with the results obtained by [1,7,33]. As for carbohydrates, their content is higher in taro flour (78.98 g/100 g) compared to wheat flour (75.58 g/100 g), which is reflected in composite flours and biscuits, with a slight increase of up to 61.73% in TWC3 (biscuit with 30% taro) compared to 60.41% in CC (control biscuit). These results corroborate those of author authors [33,34]. However, in other studies, wheat flour had a higher carbohydrate content than taro flour [1,7]. This could be explained by the origin of the taro, the methods used to process it into flour, and the storage conditions.

Incorporating taro flour into biscuit formulations would increase the mineral and carbohydrate content while reducing moisture and protein in a controlled manner. These results suggest that using up to 30% taro flour in biscuits is an interesting strategy for developing baked goods without compromising their nutritional properties.

### 3.2. Macro- and Microelements Contents in Different Samples of Flour and Cookies

**Figure 4:**
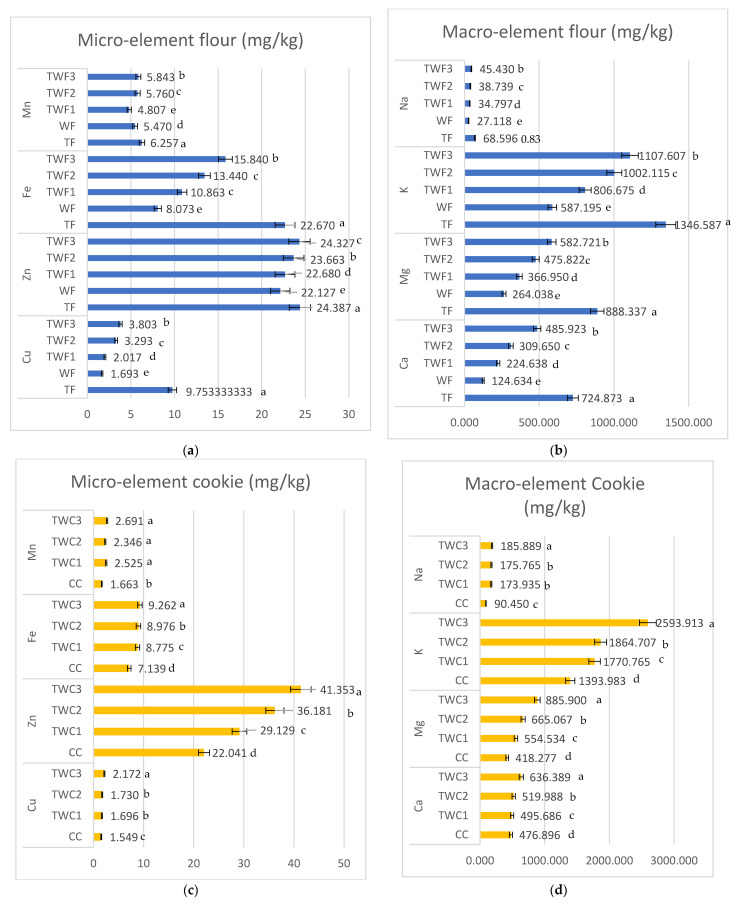
Composition of microelements and macroelements (mg/kg) in different types of flour (**a**,**b**), and in different types of cookies (**c**,**d**). The values are expressed as mean values ± standard deviations of all measurements; data sharing different letters in the same column are significantly different (*p* < 0.05), according to the Duncan test.

Figure 4 shows the microelement and macroelement composition of the different types of flour and biscuits. Analysis of these different results reveals that for all the microelements (Mn, Fe, Zn, Cu) and macroelements (Na, K, Mg, Ca) studied in this study, taro flour (TF) has significantly higher levels than wheat flour (WF). Indeed, taro flour had 6.257; 22.670; 24.387; 9.753; 68.596; 1346.587; 888.337; and 724.873 mg/kg for Mn, Fe, Zn, Cu, Na, K, Mg, and Ca, respectively. This confirms the richness of taro in essential minerals. A similar conclusion was reached by [35]. In their study entitled “Starch-based spherical aggregates: stability of a model flavouring compound, vanillin entrapped therein”, ref. [35] reported that taro flour is a good source of Fe, P, Zn, K, Cu, and Mn.

Analysis of the results also reveals that the gradual incorporation of taro flour into composite flours leads to a significant increase in most of these elements, particularly iron (10.86–15.84 mg/kg compared to 8.07 mg/kg for WF), potassium (806.68–1107. 61 mg/kg compared to 587.20 mg/kg for WF), magnesium (366.95–582.72 mg/kg compared to 264.04 mg/kg for WF) and calcium (224.64–485.92 mg/kg compared to 124.63 mg/kg for WF). Similar observations were made for cookies, where those with significant amounts of taro were the richest. These different results are consistent with those of [34,36], revealing that partially replacing wheat flour (WF) with taro flour (TF) improves the nutritional quality of composite flours and cookies. It should also be noted that these enrichments are significant from a nutritional point of view, as they concern nutrients that are often deficient in the global diet and are linked to the prevention of anemia (Fe), blood pressure regulation (K), muscle function (Mg), and bone health (Ca) [37,38,39,40]. In addition, the favorable K/Na ratio observed in composite flours and biscuits would be beneficial for cardiovascular health. Thus, the use of taro as a functional ingredient in wheat-based mixtures appears to be a promising strategy for improving the nutritional quality of cereal products, while contributing to dietary diversification and the fight against mineral deficiencies in developing countries [41,42].

### 3.3. Phytochemical Profile

#### 3.3.1. Total Polyphenol Composition of Different Flours and Biscuits Samples

**Figure 5:**
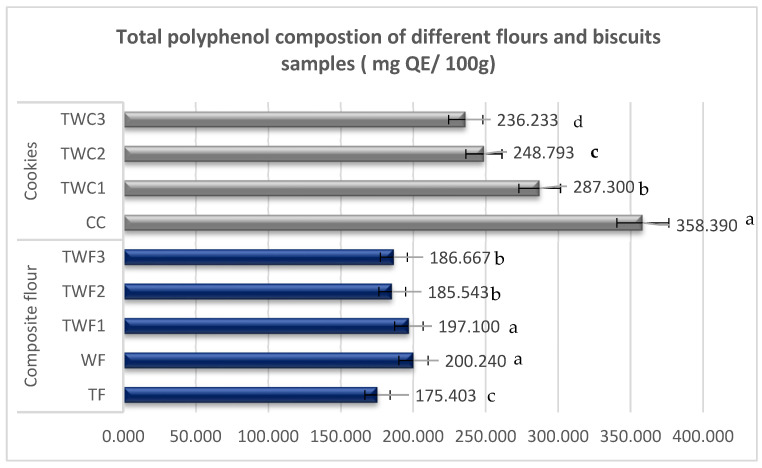
Total polyphenol composition of different flour and biscuit samples. The values are expressed as mean values ± standard deviations of all measurements; data sharing different letters in the same column are significantly different (*p* < 0.05), according to the Duncan test.

The effect of partially replacing wheat flour with taro flour on total polyphenol composition was evaluated. The results obtained (in GAE/100 g) are shown in the Figure 5.

Analysis of the total polyphenol content reveals significant variations between flours and cookies, depending on the proportion of taro and wheat used. This analysis shows that wheat flour (200.24 GAE/100 g) and TWF1 composite flour (197.10 GAE/100 g) have the highest levels, followed by TWF2 (185.54 mg QE/100 g) and TWF3 (186.67 GAE/100 g). Pure taro flour (TF) had the lowest value (175.40 GAE/100 g). These results suggest that gradually replacing wheat with taro flour could lead to a modest decrease in the polyphenol content of the mixtures. This observation is consistent with the results of previous research indicating that cereals, and wheat in particular, are a significant source of polyphenols, especially phenolic acids [43,44]. In contrast, tubers are generally lower in these compounds [43,44].

As for cookies, the polyphenol content shows a trend comparable to that observed in flours. Analysis of the data reveals that the control cookie (CC), made exclusively from wheat flour, has the highest concentration of phenolic compounds (358.39 mg QE/100 g). It is followed by TWC1 (287.30 GAE/100 g), TWC2 (248.79 GAE/100 g), and TWC3 (236.23 GAE/100 g). The gradual decrease in polyphenol concentration, concomitant with the increase in the proportion of taro in the composition, corroborates the hypothesis that TF contributes less to the supply of phenolic compounds than WF. It is interesting to note that despite the decrease in total polyphenols linked to the incorporation of taro, the enriched cookies retain significant levels (between 236.23 and 287.3 GAE/100 g). These concentrations remain adequate to confer significant antioxidant activity, given that polyphenols play a key role in protecting against oxidative stress and preventing chronic diseases [45,46]. Consequently, even though partially replacing wheat with taro reduces the polyphenol content, it also enriches the mineral content, as observed in previous analyses, which is an interesting nutritional compromise for the development of functional products.

#### 3.3.2. Individual Polyphenols in Different Flours (WF, TF, TWF1, TWF2, and TWF3) Determined by HPLC

The quantification of some phenolic compounds in WF, TF, TWF1, TWF2, and TWF3 is shown in Table 5.

Analysis of the phenolic compounds in the table above reveals significant qualitative and quantitative variability between TF, WF, and the various composite flours (TWF1, TWF2, and TWF3). Overall, taro flour (TF) is characterized by a significantly higher concentration of gallic acid (146.72 ± 1.52 mg/kg) compared to wheat flour (27.95 ± 0.17 mg/kg). This difference confirms that taro is an important source of water-soluble phenolic acids, which are known for their antioxidant activity and protective properties against oxidative stress [45,47]. In terms of flavonoids, wheat flour has a significantly higher quercetin content (20.61 ± 0.11 mg/kg) than taro flour (7.39 ± 0.22 mg/kg). However, TF has significantly higher levels of epicatechin (22 ± 0.56 mg/kg) than wheat (3.08 ± 0.15 mg/kg). Epicatechin, whose cardioprotective and anti-inflammatory effects have been demonstrated in several studies [48], may give taro significant functional potential. No statistically significant differences were observed between WF and TF in terms of hydroxycinnamic acids (caffeic acid, ferulic acid, coumaric acid, and rosmarinic acid). TF obtained 4.76 ± 0.19, 3.9 ± 0.09, 3.51 ± 0.08, and 1.43 ± 0.16 mg/kg for caffeic acid, ferulic acid, coumaric acid, and rosmarinic acid, respectively, while WF obtained 4.66 ± 0.05, 3.83 ± 0.08, 3.48 ± 0.02, and 1.58 ± 0.07 mg/kg, respectively.

The results for the composite flours indicate intermediate values, reflecting the dilution effect between the two sources (TF and WF). Thus, samples TWF1 to TWF3 retain relatively high levels of gallic acid (58.97–67.21 mg/kg) thanks to the contribution of TF, while also having significant quercetin content (16.09–17.27 mg/kg) inherited from WF. This complementarity highlights the nutritional and functional potential of blends, which balance the specific polyphenol content of the two raw materials. One of the results obtained deserves particular attention. Analyses revealed the presence of resveratrol, a natural phenolic compound, only in flours containing taro, and absent in wheat flour. This compound, which has been the subject of numerous studies due to its cardioprotective and anti-aging properties [49], has therapeutic effects on human health. In addition, the analysis reveals a residual presence of rosmarinic acid in all samples, with no significant differences between TF (1.43 ± 0.16 mg/kg) and WF (1.58 ± 0.07). Although its prevalence is low, this compound is known for its anti-inflammatory and neuroprotective properties [50] and may contribute cumulatively to the overall antioxidant effect observed in composite flours. These observations validate that the combination of taro and wheat flour would be a relevant functional alternative, due to its richness in phenolic compounds with synergistic effects.

#### 3.3.3. Antioxidant Activity

**Figure 6:**
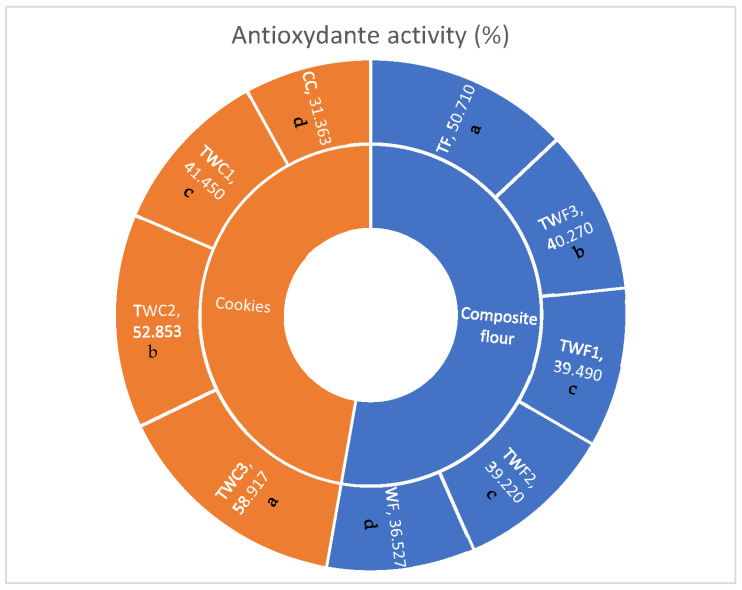
Antioxidant activity of different types of flour and biscuits. The values are expressed as mean values ± standard deviations of all measurements; data sharing different letters in the same column are significantly different (*p* < 0.05), according to the Duncan test.

The evaluation of antioxidant activity (Figure 6) highlights significant differences between flours and cookies, depending on the proportion of taro and wheat used in the formulation. Among the different varieties of flour, taro flour (TF) has the highest value (50.71%), significantly higher than that of wheat flour (WF, 36.53%). Analyses conducted on composite flours reveal intermediate activities ranging from 39.22% (TWF2) to 40.27% (TWF3). These results confirm that taro is a natural source of bioactive compounds with antioxidant potential. However, the values observed in the mixtures are slightly lower than those in pure taro. This decrease is probably due to the dilution effect of wheat flour, which is less rich in antioxidant compounds [44,51].

As for the cookies, (CC) has the lowest value (31.36%), reflecting the low antioxidant compound content of wheat flour. However, the gradual incorporation of taro significantly improves the antioxidant activity of the cookies, from 41.45% for TWC1 to 58.92% for TWC3. This increase reflects the positive contribution of taro in terms of heat-resistant bioactive compounds, such as certain flavonoids and phenolic acids, which can maintain antioxidant activity even after baking [45,46]. These results highlight an interesting paradox: although taro-enriched cookies have lower total polyphenol contents than the control cookie (CC), the antioxidant activity measured in taro-enriched cookies is significantly higher than in the wheat-based control. This suggests that the qualitative nature of polyphenols and the presence of other bioactive constituents of taro (e.g., anthocyanins and carotenoids) play a key role in overall antioxidant capacity, independent of total polyphenol content [43,44]. From a nutritional and functional perspective, incorporating taro flour into cookies appears to be a promising strategy for improving their antioxidant potential as part of a diet that prevents oxidative stress and chronic noncommunicable diseases.

### 3.4. Rheological Analysis

#### 3.4.1. Mixolab Profiler Index

The results obtained for the Mixolab profiler index for wheat flour and for the various composite flours are shown in the figure below.

**Figure 7:**
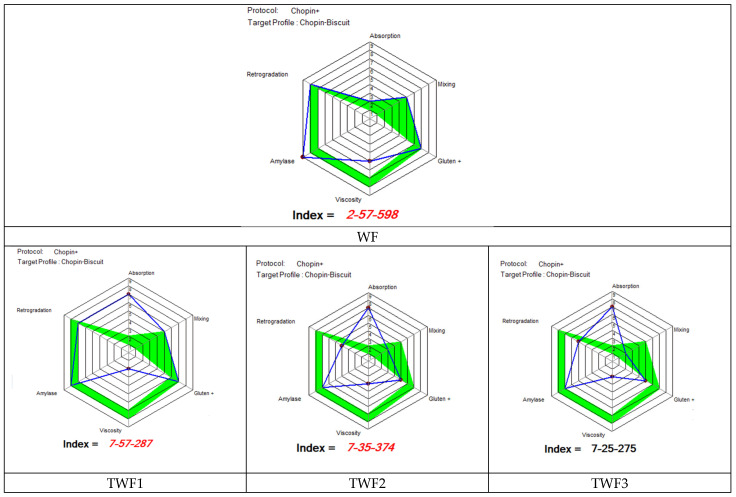
MIXOLAB Profiler index of the wheat flour type 000 partially substituted with taro flour. WF, TWF1, TWF2, and TWF3: wheat flour partially substituted with taro flour at 0%, 10%, 200%, and 30%, respectively. The blue line represents the profile of partially substituted wheat flours, and the green line represents the profile of optimal MIXOLAB parameters for cookie-making technology.

Figure 7 shows the different quality indices of the various flours studied in this study (WF, TWF1, TWF2, and TWF3) compared to the standard index for making a good biscuit using Mixolab [17]. Analysis of the results obtained from this figure reveals that the water absorption index was 2 for WF and 7 for each of the composite flours. These results show that taro flour increases the water absorption index in composite flours. These results confirm our previous findings (Section 3.1) showing low moisture content in taro flour (TF) and composite flours (TWF1, TWF2, and TWF3) compared to wheat flour (WF). The analysis of the results also shows that only WF had a water absorption index within the range (between 1 and 2) required by Mixolab for biscuit formulation. Given that the water absorption index of flour is related to the water capacity of its dough [52], it follows that doughs made from different composite flours (TWF1, TWF2, and TWF3) would have a high water absorption capacity and would therefore require special attention when formulating biscuits.

The mixing index, which is a rheological parameter that provides key information on dough stability, development time, and weakening during kneading at a temperature of 30 °C [17]. The values obtained were 5, 5, 3, and 2 for WF, TWF1, TWF2, and TWF3, respectively. This shows that substituting up to 10% of wheat flour with taro flour would have no impact on the mixing index, but a substitution of between 20% and 30% would lead to a decrease in this index. Although the mixing index was affected by high substitutions (between 20 and 30%) of wheat with taro, all the samples analyzed had an acceptable value for biscuit production according to Mixolab. According to Mixolab, a normal biscuit flour should have a mixing index between 1 and 5.

Regarding the gluten index, only samples WF (7) and TWF1 (7) obtained indices within the range required by Mixolab for a good biscuit (between 6 and 7). The other two biscuits, i.e., those with 20% (TWF2) and 30% (TWF3), obtained the same index (5) but lower than those of WF and TWF1. These results suggest that substituting up to 10% of wheat with taro has no effect on the gluten index, but substituting 20% or more reduces it. Given that the gluten index is the parameter that provides information on the behavior of gluten during dough heating, but also on the high resistance of gluten to heat when its value is high [17], replacing more than 20% of wheat with taro would produce dough with low gluten heat resistance. The retrogradation index behaved in the same way as the gluten index. In fact, only samples WF (8) and TWF1 (7) obtained values within the range required by Mixolab for a good biscuit (between 7 and 8). However, for the viscosity index, none of the flours studied in this study obtained a score suitable for biscuit production. Nevertheless, WF (5) obtained a better score than the composite flours. These results reveal that partial substitution of WF with TF would reduce the viscosity of dough during heating [17,52]. The amylase index, which is the parameter that indicates the ability of starch to resist amylolysis [17,52], behaved in the same way as the viscosity index, revealing that partial substitution of WF with TF reduces the resistance of starch to amylolysis in doughs obtained from composite flours. Nevertheless, only the composite flour had the amylase indices recommended by Mixolab for biscuit production. These results therefore reveal that the partial substitution of WF by TF, although reducing the starch’s ability to resist amylase, would nevertheless make it possible to obtain biscuit doughs that are recommended from the point of view of the amylase index.

#### 3.4.2. Dough Stability Time

**Figure 8:**
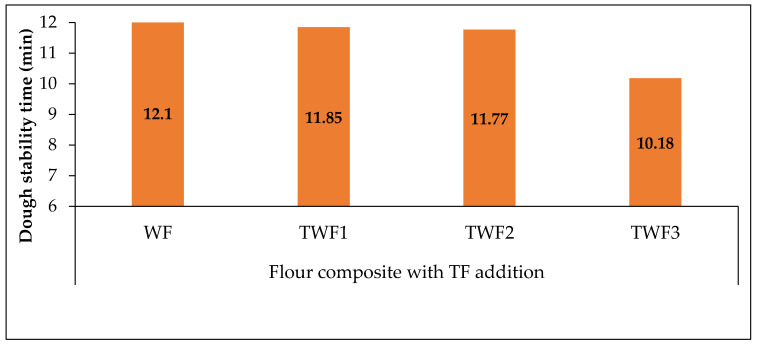
The evolution of dough stability time as a function of different composite flour formulations incorporating taro flour (TF) into wheat flour (WF).

The Figure 8 below illustrates the evolution of dough stability time as a function of different composite flour formulations incorporating taro flour (TF) into wheat flour (WF).

The stability time of the dough corresponds to the length of time during which the dough retains optimal resistance under mechanical agitation, reflecting its ability to form a stable protein network, which is largely dependent on gluten content [17]. The results show that WF has the highest stability with a time of 12.1 min. The addition of 10% taro flour (TWF1) slightly reduces this time to 11.85 min, while the addition of 20% (TWF2) reduces it to 11.77 min. These decreases are relatively small, suggesting that the gluten network is still sufficiently preserved at these levels of incorporation. In contrast, the TWF3 sample, containing 30% taro flour, showed a marked reduction in stability time, reaching 10.18 min. These values indicate that the TWF1 and TWF2 mixtures retain a consistency close to that of pure wheat, while TWF3 shows a noticeable alteration in the dough structure. This trend could be explained mainly by the dilution of gluten and the introduction of non-gluten constituents (fibers, tuber starches, soluble compounds) present in taro flour. Gluten forms the elastic network responsible for cohesion and resistance to deformation; its weakening reduces the stability measured at Mixolab.

### 3.5. Physical Analyses Performed on the Biscuits

In order to determine the impact of partially replacing wheat flour with taro flour on the technological characteristics of the biscuit, the following parameters were analyzed: thickness increase, diameter increase, baking yield, and spread ratio (D/T). The results obtained are presented in Table 6.

Analysis of the results in the table above revealed that adding taro flour to the cookie formulation led to significant changes in their technological parameters. The increase in cookie thickness, which is an indicator of its ability to rise during baking, gradually decreases with the substitution of wheat flour with taro flour. The control (CC) had the highest value (29.24%), while TWC3 had the lowest (18.95%). This reduction could be explained by the absence of gluten in taro flour, which limits gas retention and therefore the vertical expansion of the biscuits [53]. The gradual reduction in cookie thickness with the substitution of wheat flour with taro flour was also observed in studies by [7]. In their study entitled “Partial substitution of wheat flour with taro (Colocasia esculenta) flour on cookie quality” [7], obtained values falling from 25.6% to 16.7% between the control sample with 100% wheat flour and the sample substituted with 30% taro flour. These results are also consistent with those of [33].

Regarding the diameter of the cookies, a modest increase was observed, proportional to the increase in taro flour content, which rose from 5.93% (CC) to 6.16% (TWC3). This observation can be attributed to a decrease in dough resistance due to the absence of a structured gluten network (in taro flour), which promotes greater spreading during baking [54]. These results were also consistent with those of [7]. Statistical analysis of the data reveals that baking yield remains broadly stable between the different formulations (88.14% for CC versus 87.19% for TWC3). This consistency suggests that the incorporation of taro does not cause significant variations in water and volatile matter losses during the baking process. This observation reveals that the partial incorporation of taro flour into the dough composition does not significantly affect the overall thermal stability of the product. These results once again corroborate those of [7].

The spread ratio, a key parameter for biscuit quality, increased significantly with the incorporation of taro flour, from 10.70 (CC) to 11.88 (TWC3). Similar results were obtained by [7]. This improvement is generally perceived as positive in sensory terms, as it results in a crunchier texture and a more homogeneous surface [55]. The increase in the spread ratio can also be attributed to the gradual reduction in gluten (due to taro being gluten-free), leading to a more extensible dough and therefore increased spreading during baking [56]. These results suggest that the incorporation of taro flour has a predictable impact on the structure and texture of cookies, resulting in a decrease in thickness, an increase in diameter, and an improvement in the spread ratio. These technological advances, while potentially influencing sensory acceptability, also reveal the potential of taro flour as a functional ingredient. Indeed, it enables the diversification of cereal products while maintaining baking quality.

### 3.6. Analysis of Correlation

#### 3.6.1. Analysis of Correlation Between Nutritional, Macro and Microelements, and Phytochemical Parameters in Composite Flours with Taro Flour

**Figure 9:**
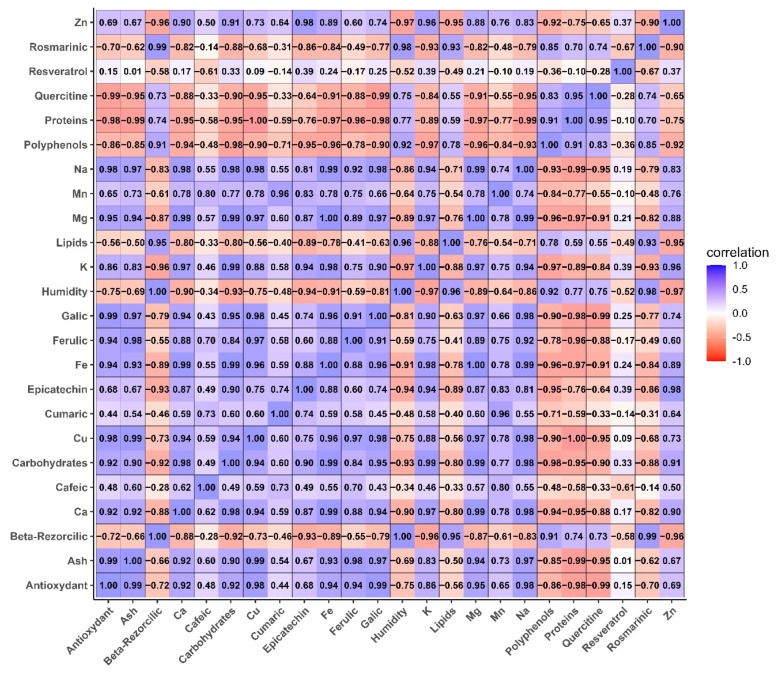
Pearson correlation between nutritional parameters, macro- and microelements, and phytochemical parameters in composite flours with different proportions of taro flour.

Figure 9 illustrate the Pearson correlations between the flours parameters analyzed. 

Correlation analysis reveals a systematic correlation between the mineral composition, phenolic compound content, and antioxidant activity of the flours studied. Parameters related to ash and macro-/microelements (Ca, Mg, K, Na, Fe, Zn, Mn) show a strong positive correlation with antioxidant activity and phenolic acids (gallic, ferulic, caffeic acid). This association suggests that adding taro flour to mixtures leads to a concomitant increase in mineral density and antioxidant potential. This observation is consistent with previous research showing that tubers are a source of both soluble minerals and phenolic metabolites, which play a role in free radical scavenging [47,51]. In this study, functional correlations were observed to highlight a link between the improved antioxidant status of the formulated products and not only the total amount of polyphenols, but also the concomitant presence of metal ions and starchy matrices. The latter modulate the stability and reactivity of phenolic compounds, as highlighted by [43].

Furthermore, the negative correlation between protein (gluten) content and antioxidant/mineral parameters reflects the protein dilution effect inherent in the substitution of wheat with taro. Indeed, increasing the proportion of taro leads to a decrease in the relative proportion of gluten, which leads to a decrease in protein indicators, while markers of mineral and bioactive richness increase. This nutritional/technological trade-off is supported by extensive scientific literature on cereal/tuber blends. Tubers have been shown to improve nutritional intake due to their richness in micronutrients and bioactive compounds. However, their incorporation into cereal/tuber mixtures also hurts the viscoelastic network formation capacity. This interaction leads to changes in the rheology and consistency of the dough [53,57].

Negative correlations have been observed between lipids and certain polyphenols. These correlations reflect intrinsic differences in matrix composition. Lipids are higher in certain cereal fractions than in the hydrophilic matrices of tubers. They are also the result of physicochemical interactions. These interactions influence the extraction and measurement of phenolic compounds. These quantitative relationships have obvious practical implications for formulation: moderate substitution (between 10 and 20%) provides nutritional benefits (minerals, polyphenols, antioxidant activity) while preserving enough protein to maintain acceptable rheological performance, as also shown by the MIXOLAB profiles and dough stability times maintained at these rates (see Figure 8). However, more substantial substitutions (30%) may highlight the nutritional benefits, but require compensatory technological approaches (protein reinforcement, hydrocolloids, hydration adjustment, or kneading optimization) to restore the consistency and malleability of the dough [55,58]. These adjustments are essential to fully exploit the functional potential of taro, while preserving industrial quality requirements and sensory acceptability criteria.

From a nutritional standpoint, the positive relationship between minerals and the antioxidant capacity of foods highlights the importance of incorporating tuber flours into everyday food products. These flours not only compensate for deficiencies in essential minerals (potassium, magnesium, calcium, and iron) but also increase the antioxidant capacity of foods. This increase in antioxidant capacity is associated with reduced oxidative stress and the prevention of chronic diseases [45].

#### 3.6.2. Analysis of Correlation Between Nutritional, Physical Analyses, Macro and Microelements, and Phytochemical Parameters in Enriched Biscuits

**Figure 10:**
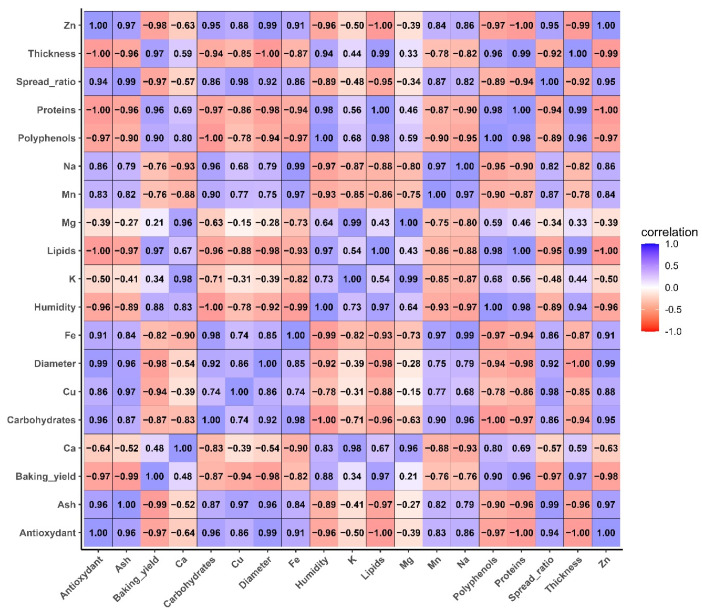
Pearson correlation between nutritional parameters, macro- and microelements, and phytochemical parameters in different types of biscuit.

Figure 10 illustrate the Pearson correlations between the biscuits parameters analyzed.

Correlation analysis for the control biscuit (CC) and those enriched with taro flour (TWC1, TWC2, and TWC3) reveals complex interactions between nutritional, functional, and technological parameters. The study results reveal a significant positive correlation between antioxidant activity and ash, polyphenol, and various mineral contents, such as Zn, Fe, Mn, Na, and Ca. These observations suggest that incorporating taro flour into biscuit production contributes to enriching them with bioactive compounds and minerals, which could enhance their antioxidant potential. This observation is consistent with previous studies that have established that polyphenol-rich tubers can increase the antioxidant value of baked goods [47].

In contrast, proteins show negative correlations with polyphenols and antioxidant activity, reflecting a gradual dilution of wheat gluten by taro flour. This substitution reduces the technological quality of the dough but improves its nutritional profile. In addition, the negative correlations between biscuit thickness and polyphenols, as well as the positive correlation between the spreading ratio and bioactive compounds, suggest that taro enrichment modifies the protein network structure and promotes spreading during baking. This observation is in line with the conclusions of the study conducted by [58], who found that reducing gluten content causes changes in the viscoelasticity and development of products.

In addition, baking yield correlates negatively with polyphenols and antioxidant activity. This suggests that increasing taro substitution may lead to reduced water retention and changes in mass loss during the baking process. These observations suggest that moderate substitution levels, between 10 and 20%, may contribute to improving the nutritional quality of cookies without significantly degrading their technological properties. On the other hand, higher proportions, reaching 30% or more, may require formulation adjustments to mitigate undesirable effects on structure and yield.

### 3.7. Principal Component Analysis and Cluster Analysis for Flours (WF, TF, TWF1, TWF2, and TWF3) and Biscuits (CC, TWC1, TWC2, and TWC3)

#### 3.7.1. Principal Component Analysis for Flours (WF, TF, TWF1, TWF2, and TWF3)

**Figure 11:**
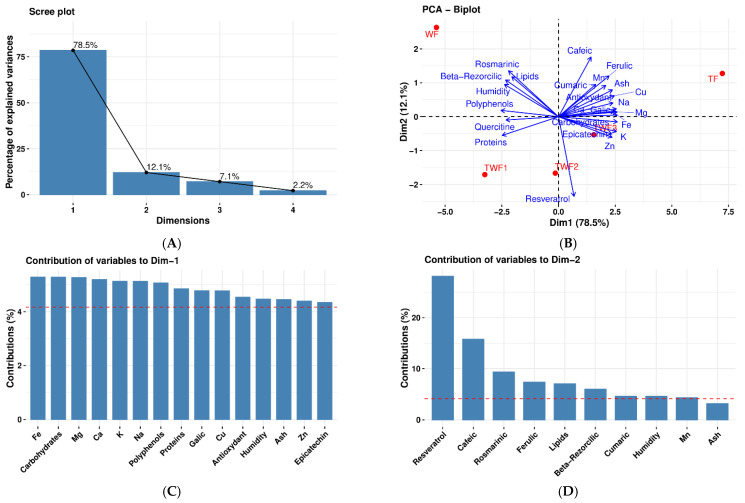
Scree plot of PCA (**A**). Biplot of PCA (**B**). Contribution of variables to the first dimension of PCA (**C**). Contribution of variables to the second dimension of PCA (**D**).

The Figure 11 shows the results of principal component analysis for the different types of flour (WF, TF, TWF1, TWF2, and TWF3).

The inertia of the first dimensions shows if there are strong relationships between variables and suggests the number of dimensions that should be studied. The first two dimensions of analysis account for 90.62% of the total dataset inertia; this means that 90.62% of the individuals (or variables) cloud’s total variability is explained by the plane (B). This percentage is very high, and thus the first plane represents the data variability very well. This value is greater than the reference value that equals 71.69%; the variability explained by this plane is thus significant (the reference value is the 0.95-quantile of the inertia percentages distribution obtained by simulating 3357 data tables of equivalent size based on a normal distribution).

The first principal component factor is significant: it expresses itself in 78.5% of the data variability (A). This axis presents an amount of inertia more significant than that obtained by the 0.95-quantile of random distributions (78.5% against 43.55%) (A) or (B). This observation suggests that this axis carries great information. The most important contribution for this component comes from the following variables: Fe, Carbohydrates, Mg, Ca, K, Na, Polyphenols, Proteins, Galic, Cu, Antioxidant, Humidity, Ash, Zn, and Epicatechin, which are highly correlated with this dimension. These variables could therefore summarize dimension 1 (C).

The second principal component expresses 12.1% of the data variability (A) or (B). The most important contribution of this component comes from the variable Resveratrol, Cafeic, Rosmarinic, Ferulic, Lipids, Beta-Rezorcilic, Cumaric, and Mn (which are all highly correlated) (D).

#### 3.7.2. Cluster Analysis for Flours (WF, TF, TWF1, TWF2, and TWF3)

**Figure 12:**
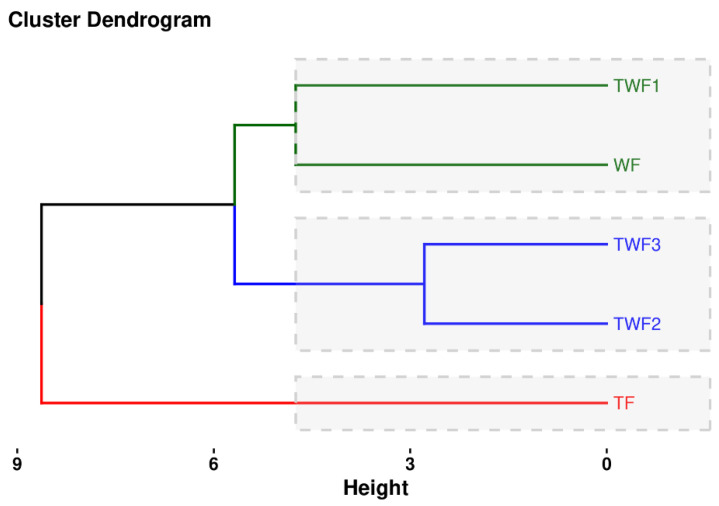
Cluster analysis for flours (WF, TF, TWF1, TWF2, and TWF3).

Cluster analysis is presented in Figure 12. This highlights the clustering of flours according to the studied variables in 3 clusters as follows: (1) Cluster 1 includes TWF1 and WF. (2) Cluster 2 includes TWF2 and TWF2. (3) Cluster 3 includes TF.

Figure 12 shows the dendrogram obtained by hierarchical classification analysis (HCA), which distinguishes three distinct groups of flours based on the variables studied. Data analysis reveals a substantial similarity between the WF (wheat flour) and TWF1 (90% wheat + 10% taro) samples. This observation suggests a similarity in the characteristics of the two samples, which is consistent with the initial hypotheses. This similarity can be explained by the low proportion of taro flour incorporated, which does not significantly alter the physicochemical and nutritional properties compared to pure wheat flour. Previous research has shown that low-level substitutions in wheat-based formulations generally induce marginal variations in technological and nutritional characteristics [7,59,60,61].

The second cluster, consisting of TWF2 (80% wheat and 20% taro) and TWF3 (70% wheat and 30% taro), suggests that increasing the proportion of taro in the mixtures brings these flours closer together while gradually differentiating them from wheat flour. This trend can be attributed to the increase in fiber, mineral, and bioactive compound concentrations, as well as the relative decrease in starch and gluten, thereby altering the functional properties of the mixtures [62,63]. The combination of TWF2 and TWF3 highlights the internal consistency of these intermediate formulations, which stand out from wheat flour while retaining a certain similarity between them.

In this study, the third cluster, consisting exclusively of TF (100% taro flour), shows a significant differentiation from all other samples analyzed. This distinction reflects the distinct nutritional and technological characteristics of taro, which is rich in insoluble fiber, minerals (potassium, magnesium), and bioactive compounds, while being gluten-free. This property gives taro flour unique properties compared to wheat flour [57].

These results confirm the effectiveness of HCA in distinguishing and classifying raw materials according to their functional and nutritional similarities [64]. From a practical standpoint, the fact that WF and TWF1 belong to the same cluster suggests that minor substitutions of taro flour can preserve properties similar to those of wheat. In contrast, TWF2 and TWF3 represent hybrid formulations with an enriched nutritional profile and differentiated functional potential. The isolation of TF reveals its value as a distinct ingredient for designing innovative formulations, particularly in the production of gluten-free products or those intended for specific nutritional purposes.

#### 3.7.3. Principal Component Analysis for Biscuits (CC, TWC1, TWC2, and TWC3)

**Figure 13:**
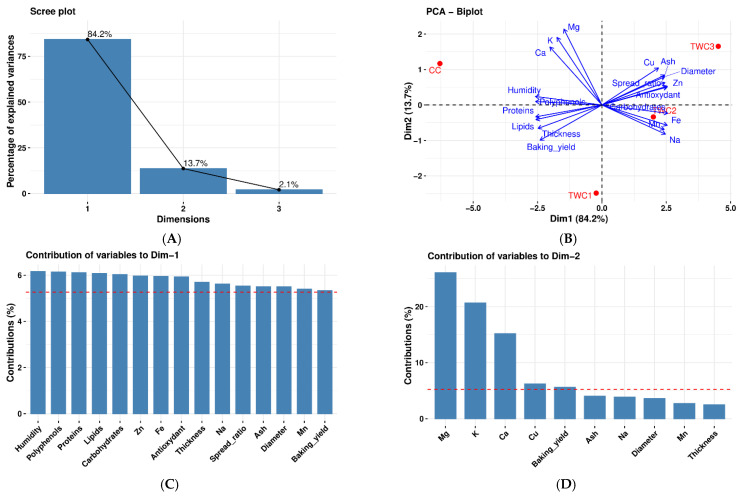
Scree plot of PCA (**A**). Biplot of PCA (**B**). Contribution of variables to the first dimension of PCA (**C**). Contribution of variables to the second dimension of PCA (**D**).

The Figure 13 shows the results of principal component analysis for the different types of biscuits (CC, TWC1, TWC2, and TWC3).

The inertia of the first dimensions shows if there are strong relationships between variables and suggests the number of dimensions that should be studied.

The first two dimensions of analysis account for 97.9% of the total dataset inertia; that means that 97.9% of the individuals (or variables) cloud’s total variability is explained by the plane (B). This percentage is very high, and thus the first plane represents the data variability very well. This value is greater than the reference value that equals 86.13%; the variability explained by this plane is thus significant (the reference value is the 0.95-quantile of the inertia percentages distribution obtained by simulating 6445 data tables of equivalent size based on a standard distribution).

The first principal component is largely dominant: it expresses itself in 84.22% of the data variability (A). This axis presents an amount of inertia more significant than that obtained by the 0.95-quantile of random distributions (84.22% against 55.49%) (A) or (B). This observation suggests that this axis carries great information. The most important contribution for this component comes from the following variables: Humidity, Polyphenols, Proteins, Lipids, Carbohydrates, Zn, Fe, Antioxidant, Thickness, Na, spread ratio, Ash Diameter, Mn, and baking yield, which are highly correlated with this dimension. These variables could therefore summarize dimension 1 (C).

The second principal component expresses 13.7% of the data variability (A) or (B). The most important contribution of this component comes from the variables Mg, K, Ca, and Cu, which are all highly correlated with this dimension (D).

#### 3.7.4. Cluster Analysis for Biscuits (CC, TWC1, TWC2, and TWC3)

**Figure 14:**
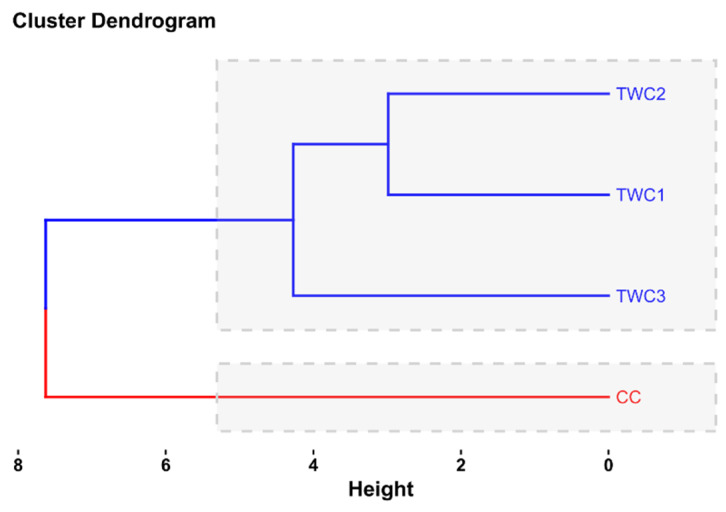
Cluster analysis for biscuits (CC, TWC1, TWC2, and TWC3).

Cluster analysis is presented in Figure 14. This highlights the clustering of biscuits according to the studied variables in 2 clusters as follows: (1) Cluster 1 includes TWC1, TWC2, and TWC3. (2) Cluster 2 includes CC.

Figure 14 illustrates the dendrogram obtained by hierarchical classification analysis (HCA), which allows cookies to be grouped according to the variables studied. Analysis of the data revealed two main clusters. The first category includes products TWC1, TWC2, and TWC3, which are cookies made by gradually increasing the proportion of taro flour (10%, 20%, and 30%) to replace traditional wheat flour. This analysis highlights the similarity between these formulations, indicating that the gradual incorporation of taro flour does not cause any significant changes to the overall profile of the cookies. However, it establishes functional and nutritional consistency between them. This observation can be attributed to a relatively similar chemical composition, particularly in terms of protein, dietary fiber, and techno-functional properties, which tend to evolve in a common direction as the proportion of taro flour increases [65].

The second cluster, consisting exclusively of CC (cookies made from 100% wheat flour), shows significant differentiation from cookies containing taro flour. This observation reflects the structural and nutritional differences between pure wheat flour and wheat–taro blends. The addition of taro flour is known to enrich the flour with fiber, minerals, and bioactive compounds, while also altering the starch content and protein profile. These processes are responsible for the separation observed in the analyses [57]. In addition, the addition of taro flour to the composition of cookies modifies several of their physical and chemical properties, including their texture, water retention capacity, and degree of browning. These changes have a significant impact on their nutritional profile, further distinguishing them from traditional recipes [7,66].

These results confirm the relevance of HCA in highlighting similarities and differences between food formulations. This relevance is consistent with previous studies that have applied multivariate methods to characterize cookies and cereal products [64,67]. From a practical standpoint, the fact that taro-enriched cookies belong to the same homogeneous group suggests a certain technological flexibility, allowing the proportions of taro to be adjusted while maintaining consistent quality. At the same time, the isolation of the 100% wheat-based biscuit reflects its distinct profile, which could be promoted for traditional preferences. In addition, taro- and wheat-based formulations offer differentiated nutritional alternatives, responding to a growing demand for products with added value in terms of fiber and bioactive compounds.

### 3.8. Spectroscopy Analysis (FTIR) of Fortified Flours with Taro Flour

**Figure 15:**
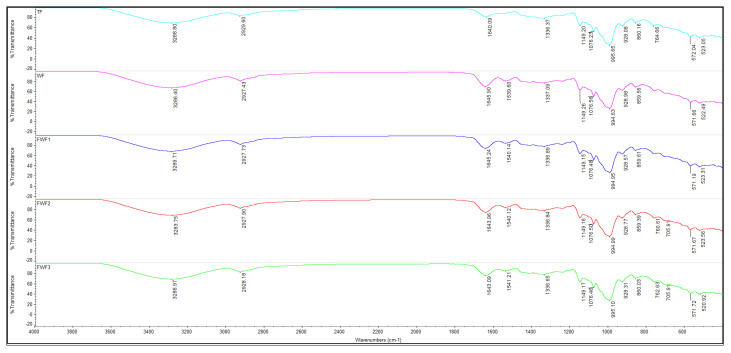
The FTIR spectra of the wheat flour type 000 partially substituted with taro flour. WF, TWF1, TWF2, and TWF3: wheat flour partially substituted with taro flour at 0%, 10%, 200%, and 30%, respectively.

The FTIR spectra of composite flours obtained after fortification of wheat flour with different percentages of taro flour are presented in Figure 15.

The results obtained showed that wheat flour is the most homogeneous sample, with an intact gluten network, which tends to provide a uniform structure and a “soft”/less brittle texture when broken. The absence of particles or fibers from taro contributes to a reduction in the micro-heterogeneity visible in the image. The scientific literature [68] highlights a key process: the dilution of gluten with TF.

As the substitution of wheat flour with taro flour intensifies, the protein matrix (gluten) becomes diluted, while the carbohydrate fraction (starch + fibers) and hydroxyl groups increase. In Fourier transform infrared spectroscopy (FTIR), these changes are manifested by the appearance of a broad O-H region (approximately 3600–3000 cm^−1^, with a maximum of approximately 3300 cm^−1^), a more significant intensity and bandwidth of the carbon region (CC) in the thermal region (TWF3), reflecting an increase in the presence of -OH groups from starch and fiber, as well as an increased proportion of bound water. In comparable starchy systems, the increase in polysaccharide concentration and hydration contributes to the strengthening of the O-H band [69]. In the aliphatic C-H region (around 2920 and 2850 cm^−1^), the trend is relatively stable between samples, with minor variations related to the lipids in the recipe and the aliphatic chains of the polysaccharides. In the study conducted by [70], it was observed that, in the context of cookies enriched with plant ingredients, these bands remain relevant indicators of the lipid/cellulose phase.

The band of triglyceride carbonyl esters (approximately 1740–1745 cm^−1^) was detected in all of the samples analyzed. In the analysis of pastries and cookies, it has been established that the classic signature of lipid esters is at 1740 cm^−1^ [71]. In the 1650–1540 cm^−1^ region, there is a gradual decrease in the relative intensity of CC in TWF3, as the taro flour dilutes the gluten. In wheat flour or dough, amide I (C=O stretching) and amide II (N–H bending + C–N stretching) are markers of the protein fraction and secondary structure; their relative decrease indicates a reduction in the contribution of proteins to the spectrum [72]. The carbohydrate fingerprint (approximately 1200 to 900 cm^−1^) and starch order markers are represented by bands at approximately 1047 and 1022 cm^−1^: the 1047/1022 ratio is commonly used as an indicator of the order (crystalline or amorphous) of starch; cooking and gelatinization tend to decrease this ratio (the more amorphous the starch, the lower the ratio).

Regarding CC, samples containing taro (TWF1-TWF3) have a higher carbohydrate contribution (the area of the 1200–900 cm^−1^ zone increases) and may show changes in the 1047/1022 ratio. In many starchy systems, this ratio decreases with thermally induced disorder; however, the origin of the starch (taro or wheat) may subtly influence this ratio [73]. The band at 995 cm^−1^ is related to the C–O–H modes of hydrated starch; a relative intensification is consistent with a greater amount of bound water and starch–water interactions in samples with TF.

### 3.9. Antinutritional Factors

In order to assess the influence of antinutritional factors on different flours and cookies, the phytic acid and oxalate content were determined for each sample. The results are presented in the Table 7.

Analysis of the results reveals that the phytic acid content varies significantly (*p* < 0.05) between different flours and cookies. Wheat flour (WF) has the highest phytic acid content (204.87 ± 1.26 mg/100 g), while taro flour (TF) has the lowest value (119.4 ± 0.46 mg/100 g). The values obtained in this study for TF were consistent with those obtained by [74] (between 31.17 and 161.13 mg/100 g) and [75] (between 17.4 and 135.3 mg/100 g). However, our results were higher than those obtained by [76], whose phytic acid levels ranged from 6.28 to 28.9 mg/100 g. The disparity in the results obtained in the different studies can be attributed to differences between the taro varieties studied, as well as to environmental conditions, which can vary considerably from one sample to another [76]. With regard to wheat flour, our results were consistent with those obtained by [77]. In their study entitled “Phytic Acid Level in Wheat Flours”, ref. [77] reported phytic acid levels ranging from 200 to 400 mg/100 g for refined wheat flours. The gradual incorporation of taro flour into wheat flour (TWF1 to TWF3) resulted in a significant reduction in phytic acid levels, from 190.52 ± 0.36 mg/100 g (TWF1) to 153.43 ± 1.37 mg/100 g (TWF3). This trend indicates that partially replacing wheat with taro flour helps reduce the phytic acid content, confirming the dilutive effect of taro flour, which is lower in phytates. A similar trend was observed for cookies. The phytic acid content of the wheat-based control cookie (CC) was 192.49 ± 0.14 mg/100 g, whereas that of the cookies containing taro decreased gradually with increasing proportions of taro flour, reaching 134.33 ± 0.52 mg/100 g for TWC3. This decrease can be attributed not only to the lower initial phytate content of TF compared to WF, but also to the effect of heat treatment during baking, which is likely to hydrolyze or complex some of the phytic acids [78]. It is important to note that the phytic acid content detected in the different flours and cookies in this study is below the maximum acceptable dose for the body, generally ranging between 250 and 500 mg/100 g [76,79].

Regarding oxalate, the values obtained exhibit an inverse trend compared to those for phytic acid. TF (66.73 ± 0.67 mg/100 g) contains significantly higher levels of oxalate than wheat flour (22.15 ± 0.67 mg/100 g). Our results for TF were consistent with those of [80,81] (between 65 and 171 mg/100 g). However, they were lower than those obtained by [82] (70.5 and 218.8 mg/100 g) and higher than those obtained by [76] (24.3 and 56.7 mg/100 g). The observed discrepancy in the results obtained in the different studies can be attributed to differences between the taro varieties studied, as well as to environmental conditions, which can vary considerably from one sample to another [76]. In composite flours, the increasing incorporation of taro logically leads to an increase in the oxalate content, from 34.1 ± 1.1 mg/100 g (TWF1) to 39.89 ± 0.34 mg/100 g (TWF3). Although taro flour enrichment improves certain nutritional aspects, it can slightly increase the oxalic load of the final product, which may reduce the bioavailability of calcium and iron [83,84]. The same pattern is observed for cookies: the control cookie (CC) contains 20.61 ± 0.67 mg/100 g of oxalate, while the level rises to 37.99 ± 0.83 mg/100 g for the cookie containing 30% taro flour (TWC3). However, this increase remains moderate compared to the values observed in flours, probably due to the partial thermal degradation of oxalates during baking [81]. It is also important to note that although taro contributes to the increase in oxalate levels in different flours and biscuits, their consumption does not pose any risk to consumers, given that toxic and lethal oxalate levels for humans are between 3 and 5 g [76,79].

### 3.10. Sensory Analysis

**Figure 16:**
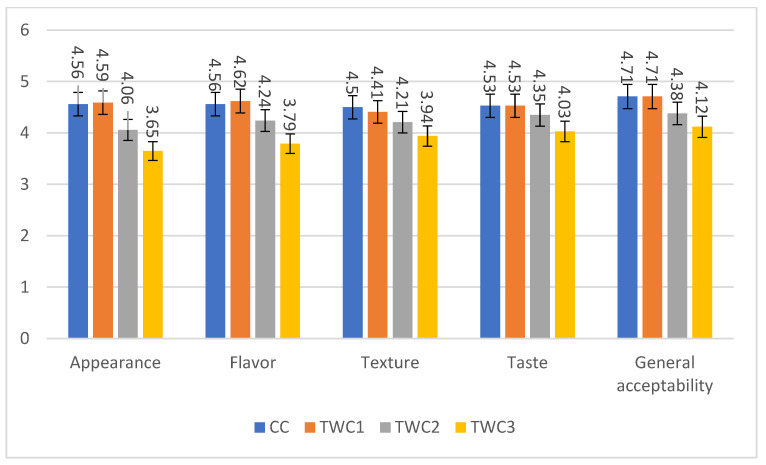
Consumer acceptance of biscuits with different taro flour proportions, using a 5-point hedonic scale (*n* = 34). Mean values of appearance, flavor, texture, taste, and general acceptability.

The impact of enriching wheat-based cookies with taro on sensory quality was studied. The results are presented in the Figure 16.

The results of the sensory analysis of cookies formulated with different levels of taro flour substitution (10%, 20%, and 30%) reveal a clear trend: moderate substitution (10%) improves or maintains sensory quality, while higher substitution (20% and 30%) tends to decrease overall acceptability. Similar results have been obtained by [1,7,33]. In terms of appearance, the control cookie (CC) and the TWC1 cookie obtained the highest scores (4.56 and 4.59, respectively), showing that the incorporation of 10% taro flour does not alter the visual appearance of the product. In contrast, the scores gradually decreased with increasing substitution levels (TWC2: 4.06; TWC3: 3.65), which could be attributed to a darker and less homogeneous coloration linked to the presence of pigments and fibers in taro flour, as observed in other studies on root flours [61]. Results showing similar behavior in cookies were also obtained by [7,33].

Flavor and taste follow a similar trend: TWC1 cookies received the highest scores (4.62 and 4.53), higher than those of the control (CC), suggesting that the addition of low doses of taro enriches the sensory profile, probably due to the presence of specific aromatic compounds. However, higher substitution levels resulted in a significant decrease (TWC2: 4.24 and 4.35; TWC3: 3.79 and 4.03). These results were consistent with those of [7]. In terms of texture, the control and TWC1 were rated most satisfactory (4.5 and 4.41), while TWC3 received the lowest rating (3.94). This decrease may be related to the progressive dilution of gluten with the increase in the percentage of taro, resulting in increased crumbly texture and a less cohesive structure, as reported in similar studies on products enriched with gluten-free flours [85].

Finally, overall acceptability reflects the combination of these parameters: CC and TWC1 cookies received the highest ratings (4.71 and 4.71), revealing that incorporating 10% taro flour would be a better compromise between nutritional value, technological functionality, and sensory satisfaction. In contrast, a higher substitution (20% and 30%) significantly reduces acceptability (4.38 and 4.12), suggesting that taro should be used in limited proportions in formulations intended for everyday consumption.

## 4. Conclusions

This study demonstrated that incorporating taro flour into wheat-based cookie formulations significantly improves the nutritional and functional quality of the final product. Analysis of the results highlights the potential of taro flour as a functional and nutritional ingredient in the formulation of composite flours and cookies. The gradual incorporation of taro flour led to a notable improvement in the content of essential minerals, such as potassium (K), magnesium (Mg), zinc (Zn), iron (Fe), manganese (Mn), and calcium (Ca), as well as bioactive compounds, including phenolic acids, flavonoids, and resveratrol. This approach resulted in a significant increase in the antioxidant activity of the product. These nutritional and functional improvements confirm the benefits of partially replacing wheat with taro in the development of baked goods with higher health value. However, technological analyses revealed that replacing wheat flour with taro flour leads to a decrease in dough stability, baking yield, and specific structural properties, attributable to gluten dilution. As part of the study conducted on the antinutritional profile of flours and cookies, the analysis revealed that the addition of taro flour led to a significant reduction in phytic acid, while inducing a moderate increase in oxalate in the different flours and in the cookies. In addition, sensory evaluation revealed a gradual decrease in acceptability when the substitution level exceeded 20%, with a marked preference for biscuits enriched with 10% (TWC1), whose overall quality remained comparable to the pure wheat control. Thus, moderate substitution (10–20%) appears to be an optimal compromise between nutritional improvement and the preservation of technological and sensory qualities. This innovative approach presents promising prospects for promoting taro in functional food formulations, particularly in bakery products, while contributing to the diversification of the use of this underutilized local resource.

## Figures and Tables

**Table 1:** Different cookies obtained and the composition of the ingredients used in their formulation.

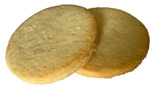		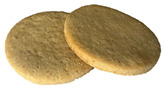		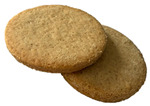		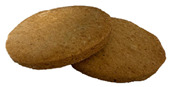	
CC		TWC1		TWC2		TWC3	
Ingredient	Quantity	Ingredient	Quantity	Ingredient	Quantity	Ingredient	Quantity
Wheat flour (WF)	0.5 kg	Wheat flour (WF)	0.5 kg	Wheat flour (WF)	0.5 kg	Wheat flour (WF)	0.5 kg
Taro flour (TF)	-	Taro flour (TF)	0.050 kg	Taro flour (TF)	0.100 kg	Taro flour (TF)	0.150 kg
Butter	0.250 kg	Butter	0.250 kg	Butter	0.250 kg	Butter	0.250 kg
Baking powder	0.008 kg	Baking powder	0.008 kg	Baking powder	0.008 kg	Baking powder	0.008 kg
Yolk	0.060 kg	Yolk	0.060 kg	Yolk	0.060 kg	Yolk	0.060 kg
Milk	20 mL	Milk	20 mL	Milk	20 mL	Milk	20 mL
Sugar	0.150 kg	Sugar	0.150 kg	Sugar	0.150 kg	Sugar	0.150 kg

**Table 2:** Analytical methods used for the determination of the nutritional composition for different samples.

Parameters	Methods	References
Moisture and protein content (%)	Analyzed using ICC Standard Methods (2003)	[20]
Ash content (%)	Determined according to ISO Method No. 2171:2007	[21]
Fat content (%)	Measured following AOAC Official Method (2000)	[22]
The carbohydrate content (g/100 g)	Calculated by difference: 100 − (moisture + ash + protein + fat)	[23]

**Table 3:** Analytical methods used for the determination of total phenolic content (TPC), antioxidant activity (AA), and individual polyphenols by HPLC.

Parameters	Samples Analyzed	Methods	Units
Total phenolic content (TPC)	All flour and cookie samples.	The total phenolic content (TPC) was determined using previously prepared alcoholic extracts, according to the Folin-Ciocâlteu method described by Danciu et al. (2018) [25] and Obistioiu et al. (2021) [26]. It should be noted that all determinations were performed in triplicate.	GAE/100 g (Milligrams of gallic acid equivalents (GAE) per 100 g)
Antioxidant activity (AA)	All flour and cookie samples.	The antioxidant activity was performed using the method described by Ciulca et al. [27] was used, with a few modifications. This method is based on the use of 2,2-diphenyl-1-picrylhydrazyl (DPPH) reagent (Sigma-Aldrich; Merck KGaA, Darmstadt, Germany). As part of the experiment, 1 mL (*v*/*v*) of alcoholic extract from each sample (1/10) and 2.5 mL of a 0.03% mM DPPH solution were mixed. The concentration of investigated extracts where 10, 5, 2.5, 1.25 and 0.67 mg/mL. The mixture was then incubated for 30 min at room temperature in a dark environment. The absorbance was then measured using a spectrophotometer (model Specord 205; Analytik Jena AG, Jena, Germany) at a wavelength of 518 Nm. Ethanol was used as a positive control in this study. Antioxidant activity was evaluated using the following equation: AA (%) = (Control Absorbance − Sample Absorbance/Control Absorbance) × 100 Where Control Absorbance refers to the absorbance values of the control, and Sample Absorbance refers to the absorbance values of the sample.	%
Individual polyphenols by HPLC	All flour samples.	In order to determine the individual levels of polyphenols in the different flour samples, the method described by [25] was used, with a few minor adjustments. In this study, a Shimadzu chromatograph was used, equipped with SPD-10A UV detector (Shimadzu, Kyoto, Japan), For the analysis, column Adsorbosphere UHS C18 5 μm, batch 007250 (Dr. Maisch, Ammerbuch, Germany) was used. The following chromatographic conditions were applied: Mobile phase A consisted of water acidified with formic acid to a pH of 3, Mobile phase B was created using a combination of acetonitrile acidified with formic acid to a pH of 3. The gradation program, which is the precise sequence of durations and intensities of light used in the photographic development process, was as follows: 0.01 to 20 min at 5% blue; 20.01 to 50 min at 5 to 40% blue; 50 to 55 min at 40 to 95% blue; 55 to 60 min at 95% blue. The solvent flow rate was measured at 0.3 mL/min at a temperature of 20 °C, while the wavelength used for the control ranged from 280 to 320 nm. The calibration curves were produced in the range of 20 to 50 μg/mL. It should be noted that all measurements were performed in triplicate.	mg/kg

**Table 4:** Proximate composition of composite flours.

Samples	Nutritional Characteristics				
	Moisture	Ash	Proteins	Lipids	Carbohydrates
	(%)	(%)	(%)	(%)	(g/100 g)
Composite flours					
TF	10.38 ± 0.02 ^e^	2.58 ± 0.02 ^a^	6.76 ± 0.02 ^e^	1.3 ± 0.01 ^b,c^	78.98 ± 0.04 ^a^
WF	11.50 ± 0.1 ^a^	0.28 ± 0.02 ^e^	11.30 ± 0.04 ^a^	1.33 ± 0.01 ^a^	75.58 ± 0.05 ^e^
TWF1	10.99 ± 0.02 ^b^	0.35 ± 0.01 ^d^	10.99 ± 0.02 ^b^	1.32 ± 0.01 ^b^	76.35 ± 0.01 ^d^
TWF2	10.73 ± 0.06 ^c^	0.48 ± 0.01 ^c^	10.33 ± 0.02 ^c^	1.31 ± 0.01 ^b,c^	77.15 ±0.07 ^c^
TWF3	10.53 ± 0.03 ^d^	0.76 ± 0.02 ^b^	9.99 ± 0.02 ^d^	1.30 ± 0.01 ^c^	77.43 ± 0.05 ^b^
Cookies					
CC	6.63 ± 0.01 ^a^	1.07 ± 0.01 ^d^	10.84 ± 0.02 ^a^	21.04 ± 0.04 ^a^	60.41 ± 0.04 ^c^
TWC1	6.14 ± 0.02 ^b^	1.27 ± 0.01 ^c^	10.40 ± 0.01 ^b^	20.94 ± 0.02 ^b^	61.24 ± 0.04 ^b^
TWC2	5.96 ± 0.04 ^c^	1.44 ± 0.03 ^b^	10.06 ± 0.03 ^c^	20.86 ± 0.02 ^c^	61.68 ± 0.01 ^a^
TWC3	5.86 ± 0.04 ^d^	1.78 ± 0.01 ^a^	9.83 ± 0.03 ^d^	20.80 ± 0.02 ^d^	61.73 ± 0.08 ^a^

The values are expressed as mean values ± standard deviations of all measurements; data sharing different letters in the same column are significantly different (*p* < 0.05), according to the Duncan test.

**Table 5:** Quantification of individual polyphenols.

Samples									
	Galic	Epicatechin	Cafeic	Beta-Rezorcilic	Cumaric	Ferulic	Rosmarinic	Resveratrol	Quercitine
	(mg/kg)	(mg/kg)	(mg/kg)	(mg/kg)	(mg/kg)	(mg/kg)	(mg/kg)	(mg/kg)	(mg/kg)
TF	146.72 ± 1.52 ^a^	22 ± 0.56 ^a^	4.76 ± 0.19 ^a^	3.63 ± 0.26 ^b^	3.51 ± 0.08 ^a^	3.9 ± 0.09 ^a^	1.43 ± 0.16 ^a^	3.49 ± 0.07 ^b^	7.39 ± 0.22 ^d^
WF	27.95 ± 0.17 ^e^	3.08 ± 0.15 ^e^	4.66 ± 0.05 ^a^	4.11 ± 0.11 ^a^	3.48 ± 0.02 ^a^	3.83 ± 0.08 ^a^	1.58 ± 0.07 ^a^	ND	20.61 ± 0.11 ^a^
TWF1	58.97 ± 1 ^d^	5.28 ± 0.3 ^d^	4.37 ± 0.19 ^a^	3.88 ± 0.09 ^a,b^	3.41 ± 0.14 ^a^	3.82 ± 0.08 ^a^	1.49 ± 0.00 ^a^	2.2 ± 0.59 ^a,b^	16.09 ± 0.13 ^c^
TWF2	62.21 ± 0.49 ^c^	17.97 ± 0.22 ^c^	4.47 ± 0.36 ^a^	3.75 ± 0.12 ^b^	3.5 ±0.24 ^a^	3.82 ± 0.07 ^a^	1.47 ±0.03 ^a^	2.71 ± 0.89 ^a^	17.04 ± 0.08 ^b^
TWF3	67.21 ± 0.77 ^b^	19.37 ± 0.14 ^b^	4.74 ± 0.26 ^a^	3.7 ± 0.08 ^b^	3.49 ± 0.04 ^a^	3.84 ± 0.02 ^a^	1.45 ± 0.01 ^a^	1.32 ± 0.19 ^b^	17.27 ± 0.06 ^b^

The values are expressed as mean values ± standard deviations of all measurements; data sharing different letters in the same column are significantly different (*p* < 0.05), according to the Duncan test.

**Table 6:** The physical parameters of biscuits.

Samples	Thickness Increase (%)	Diameter Increase (%)	Baking Yield (%)	Spread Ratio (D/T)
CC	29.24 ± 0.31 ^a^	5.93 ± 0.03 ^a^	88.14 ± 0.06 ^a^	10.7 ± 0.05 ^a^
TWC1	26.18 ± 0.12 ^b^	5.98 ± 0.03 ^b^	87.94 ± 0.06 ^b^	11.14 ± 0.01 ^b^
TWC2	21.09 ± 0.01 ^c^	6.11 ± 0.02 ^c^	87.59 ± 0.09 ^c^	11.26 ± 0.02 ^c^
TWC3	18.95 ± 0.16 ^d^	6.16 ± 0.03 ^d^	87.19 ± 0.04 ^d^	11.88 ± 0.01 ^d^

The values are expressed as mean values ± standard deviations of all measurements; data sharing different letters in the same row are significantly different (*p* < 0.05), according to the Duncan test.

**Table 7:** Antinutritional factors of different flours and cookies.

Samples	Antinutritional Characteristics	
	Total Phytic Acid	Total Oxalate
	(mg/100 g)	(mg/100 g)
Composite flours		
TF	119.4 ± 0.46 ^e^	66.73 ± 0.67 ^a^
WF	204.87 ± 1.26 ^a^	22.15 ± 0.67 ^e^
TWF1	190.52 ± 0.36 ^b^	34.1 ± 1.1 ^d^
TWF2	172.81 ± 1.44 ^c^	37.91 ± 0.55 ^c^
TWF3	153.43 ± 1.37 ^d^	39.89 ± 0.34 ^b^
Cookies		
CC	192.49 ± 0.14 ^a^	20.61 ± 0.67 ^d^
TWC1	174.21 ± 1.14 ^b^	31.97 ± 0.89 ^c^
TWC2	157.94 ± 1.15 ^c^	36.45 ± 0.71 ^b^
TWC3	134.33 ± 0.52 ^d^	37.99 ± 0.83 ^a^

The values are expressed as mean values ± standard deviations of all measurements; data sharing different letters in the same row are significantly different (*p* < 0.05), according to the Duncan test.

## Data Availability

The original contributions presented in this study are included in the article. Further inquiries can be directed to the corresponding authors.
